# Inflammatory cytokine response to exercise in alpha-1-antitrypsin deficient COPD patients ‘on’ or ‘off’ augmentation therapy

**DOI:** 10.1186/1471-2466-14-106

**Published:** 2014-06-30

**Authors:** I Mark Olfert, Moh H Malek, Tomas ML Eagan, Harrieth Wagner, Peter D Wagner

**Affiliations:** 1 Center for Cardiovascular and Respiratory Sciences, Division of Exercise Physiology, One Medical Center Dr, West Virginia University School of Medicine, Morgantown, WV 26506-9105, USA; 2 Integrative Physiology of Exercise Laboratory, Eugene Applebaum College of Pharmacy & Health Sciences, Wayne State University, 259 Mack Avenue, Detroit, MI 48201, USA; 3 Department of Medicine, Division of Physiology, University of California, San Diego, 9500 Gilman Drive, La Jolla, CA 92093-0623, USA; 4 Department of Thoracic Medicine, Haukeland University Hospital, Jonas Lies vei 65, N-5021 Bergen, Norway

**Keywords:** Tumor necrosis factor-α, C-reactive protein, Skeletal muscle, Emphysema, Inflammation

## Abstract

**Background:**

There is still limited information on systemic inflammation in alpha-1-antitrypsin-deficient (AATD) COPD patients and what effect alpha-1-antitrypsin augmentation therapy and/or exercise might have on circulating inflammatory cytokines. We hypothesized that AATD COPD patients on augmentation therapy (AATD + AUG) would have lower circulating and skeletal muscle inflammatory cytokines compared to AATD COPD patients not receiving augmentation therapy (AATD-AUG) and/or the typical non-AATD (COPD) patient. We also hypothesized that cytokine response to exercise would be lower in AATD + AUG compared to AATD-AUG or COPD subjects.

**Methods:**

Arterial and femoral venous concentration and skeletal muscle expression of TNFα, IL-6, IL-1β and CRP were measured at rest, during and up to 4-hours after 50% maximal 1-hour knee extensor exercise in all COPD patient groups, including 2 additional groups (i.e. AATD with normal lung function, and healthy age-/activity-matched controls).

**Results:**

Circulating CRP was higher in AATD + AUG (4.7 ± 1.6 mg/dL) and AATD-AUG (3.3 ± 1.2 mg/dL) compared to healthy controls (1.5 ± 0.3 mg/dL, p < 0.05), but lower in AATD compared to non-AATD-COPD patients (6.1 ± 2.6 mg/dL, p < 0.05). TNFα, IL-6 and IL-1β were significantly increased by 1.7-, 1.7-, and 4.7-fold, respectively, in non-AATD COPD compared to AATD COPD (p < 0.05), and 1.3-, 1.7-, and 2.2-fold, respectively, compared to healthy subjects (p < 0.05). Skeletal muscle TNFα was on average 3–4 fold greater in AATD-AUG compared to the other groups (p < 0.05). Exercise showed no effect on these cytokines in any of our patient groups.

**Conclusion:**

These data show that AATD COPD patients do not experience the same chronic systemic inflammation and exhibit reduced inflammation compared to non-AATD COPD patients. Augmentation therapy may help to improve muscle efflux of TNFα and reduce muscle TNFα concentration, but showed no effect on IL-6, IL-1β or CRP.

## Background

Alpha-1 antitrypsin deficiency (AATD) is an inherited genetic disorder that leads to development of emphysema resulting in chronic obstructive pulmonary disease (COPD) among other complications (e.g. liver dysfunction). The lack of functional α_1_-antitrypsin exposes the lungs to an insufficient inhibition of serine protease activity leading to chronic uncontrolled proteolytic attack that results in early-life (<40 years of age) alveolar destruction, which can occur independent of environmental risk factors, such as cigarette smoke. Since the first report identifying the connection between abnormal alpha-1 antitrypsin and COPD in 1963 [1], more than 90 different α_1_-antitrypsin phenotypes have been described. The most common protein inhibitor (Pi) variants are the M, S, and Z alleles, with the PiMM representing normal genotype and PiMS, PiSS, PiMZ, PiZS and PiZZ representing genotypes of increasing severity for abnormal circulating α_1_-antitrypsin concentration (ranging from 20% loss in PiMS to >85% loss in PiZZ individuals) [2]. The rates of decline in lung function and onset of emphysema can be highly variable in AATD individuals, in part due to phenotypic differences in α_1_-antitrypsin reductions; but also due to varying individual exposures to environmental risk factors (most notably cigarette smoke history) [3,4].

In people with normal circulating AAT levels, environmental irritants (such as cigarette smoke, coal dust, etc.) induces acute and chronic airway inflammation that plays a prominent role in development and progression of COPD [5]. Systemic inflammation is also prevalent in many patients with COPD [6,7], but it remains controversial whether systemic inflammation represents ‘spill over’ from the lung inflammation or activation of a chronic systemic process [8]. Irrespective of the source, elevated circulating levels of inflammatory biomarkers, such as tumor necrosis factor-alpha (TNFα), C-reactive protein (CRP), interleukin (IL)-6, and IL-1β have all been associated with severity and exacerbations of COPD and may contribute to comorbidities accompanying COPD [9-11]. In patients with AATD COPD, there is evidence that abnormal alleles of α_1_-antitrypsin can produce polymers that can act as neutrophil chemoattractants (such as Pi‘Z’ polymers) which may contribute to a pro-inflammatory state and development of emphysema [12,13], and is consistent with increased expression of pulmonary inflammatory mediators in AATD individuals [14-16]. In addition, PiMZ subjects without airway obstruction may also exhibit neutrophilic inflammation in their airways, and therefore at greater risk in developing pulmonary changes [15]. Indeed, native α_1_-antitrypsin (AAT) serves as an endogenous inhibitor of proinflammatory cytokine production [17,18], implying that loss or reductions in AAT concentration could make AATD individuals more susceptible to chronic systemic inflammation. Management of AATD patients with COPD can also include treatment with purified AAT to replace/augment circulating AAT levels, commonly referred to as ‘augmentation therapy’. Some evidence suggests that augmentation therapy may reduce airway inflammation [14,19-22] and potentially decrease the progression of COPD symptoms [23]. To our knowledge, however, there are no studies that have compared circulating and skeletal muscle inflammatory cytokines in AATD patients ‘on’ versus ‘off’ augmentation therapy, or in individuals with AATD with normal lung function.

Exercise is commonly prescribed as part of the medical treatment plan for patients with COPD. While the benefits of exercise to the immune system [24] and to cardiovascular health are widely recognized, exercise is also known to stimulate increases in pro-inflammatory cytokines, such as TNFα and IL-6 [25]. In healthy individuals, the magnitude and duration of these increases are off-set by similar increases in anti-inflammatory cytokines [25-27]. One concern for patients with COPD is that the combination of exercise and an already elevated circulating inflammatory cytokine level may prove more harmful than good. Previous studies have reported on the effects of exercise and expression of circulating inflammatory mediators in non-AATD patients with COPD showing either increased [28-31] or no change [32-36] in circulating cytokines with exercise. No studies, thus far, have assessed these responses to exercise in AATD individuals with or without COPD, and whether or not augmentation therapy affects the circulating and/or skeletal muscle expression of inflammatory cytokines response to exercise.

Accordingly, to better understand the potential influence of augmentation therapy on biomarkers of systemic inflammation, and the relative risk of exercise contributing to chronic systemic inflammation in AATD individuals, we examined systemic expression of several key inflammatory cytokines in AATD COPD patients receiving and not receiving augmentation therapy, as well as AATD individuals without evidence of obstructive airway disease. Our evaluation also included two control groups: non-AATD COPD patients (with normal α_1_-antitrypsin levels), and age-matched healthy control subjects. We hypothesized, under resting conditions, α_1_-antitrypsin replacement therapy in AATD patients would result in lower expression of circulating and skeletal muscle inflammatory cytokines, compared to the other AATD individuals and/or the traditional (non-AATD) COPD patients. We also hypothesized that circulating cytokine response to exercise would be lower in AATD patients receiving α_1_-antitrypsin replacement therapy compared to COPD or the other AATD subjects.

## Methods

### Subjects

AATD subjects (n = 16) were recruited by a national advertisement campaign in the US and brought to the Human Research Laboratory at the Division of Physiology, University of California, San Diego. Exclusion criteria included history of lung disease (other than COPD), cardiovascular disease, diabetes mellitus or any other metabolic diseases, cancer, oral corticosteroid use in the last 6 months, and/or respiratory tract infection within the last 4 weeks, as determined by medical history and patient’s list of medication. Presence of COPD was defined according Global Initiative for Chronic Obstructive Lung Disease (GOLD) 2009 COPD guidelines (http://www.goldcopd.org). Inclusion criteria for AATD subjects were ZZ or SZ genotype with or without history of COPD, whereas control subjects genotype were MM or MZ genotype. AATD patients were grouped into 3 categories, AATD with COPD receiving augmentation therapy (AATD + AUG, n = 6), AATD with COPD not receiving augmentation therapy (AATD–AUG, n = 6), and AATD with normal lung function (AATD_PFTnorm_, n = 4). Control COPD patients (with normal α_1_-antitrypsin concentration levels, n = 7) and healthy control subjects (n = 6) were recruited by advertisement within the Southern California region. Healthy control subjects were matched for age, sex, and claimed to not participate in regular structured exercise (i.e. running, cycling, etc.) or sport activity. All participants were screened for α_1_-antitrypsin genotyped from a blood sample and had circulating AAT concentration measured (Biocerna LLC, Gaithersburg, MD) (Table 1). All subjects, except for some in the non-AATD COPD group, indicated they were either no longer smoking (at least > 1 year) or had never smoked. Within the non-AATD COPD group, 57% of the subjects were current smokers (Table 2).

**Table 1:** Anthropometric, medication and liver function data of subject groups

	**AATD + AUG**^**a**^	**AATD + AUG**^**b**^	**TD PFTnorm**^**c**^	**non-AATD COPD**^**d**^	**Healthy**^**e**^
Lung health classification	COPD	COPD	Normal	COPD	Normal
Genotype	PiZZ	PiZZ (PiSZ = 1)	PiZZ	PiMM	PiMM (PiMZ = 1)
Number of subjects (m,f)	6 (3,3)	6 (2,4)	4 (1,3)	7 (4,3)	5 (3,2)
Age (years)	56 ± 4	63 ± 5	55 ± 5	64 ± 2	56 ± 1
Height (cm)	177 ± 3	170 ± 4	172 ± 5	167 ± 4	172 ± 5
Weight (kg)	80 ± 10	83 ± 13	77 ± 9	82 ± 8	86 ± 10
DXA body fat (%)	32 ± 4	37 ± 3	33 ± 7	30 ± 4	32 ± 5
Lean body mass (kg)	54 ± 6	52 ± 8	51 ± 7	57 ± 5	58 ± 7
Body mass Index (kg/m^2^)	25 ± 2	28 ± 3	26 ± 3	29 ± 2	29 ± 2
Fat-free index (kg/m^2^)	17 ± 1	18 ± 2	17 ± 2	20 ± 1	19 ± 1
Lean body mass (DXA) (kg)	49 ± 7	51 ± 7	48 ± 7	53 ± 5	54 ± 6
Thigh mass (DXA) (kg)	16 ± 2	13 ± 2	16 ± 2	15 ± 1	16 ± 2
**Medication**					
No prescribed meds	0%	0%	100%	14%	60%
AAT Augmentation Therapy	100%	0%	0%	0%	0%
Bronchodilator	100%	100%	0%	86%	0%
Inhaled corticosteriods	83%	83%	0%	71%	0%
Oxygen	33%	0%	0%	29%	0%
Diuretics	0%	17%	0%	14%	0%
Anti-inflammatory	0%	0%	0%	0%	0%
Anti-depressive	0%	17%	0%	29%	0%
β-blockers	33%	33%	0%	29%	40%
**Serum liver function tests**					
Total Bilirubin (mg/dL)	0.48 ± 0.06	0.50 ± 0.12	0.45 ± 0.09	0.37 ± 0.05	0.51 ± 0.12
Albumin (g/dL)	3.80 ± 0.17	3.45 ± 0.15	3.80 ± 0.12	3.77 ± 0.11	3.82 ± 0.06
Alanine Aminotransferase (U/L)	42.3 ± 15.2	28.5 ± 3.4	41.0 ± 12.7	23.4 ± 5.3	30.6 ± 2.9
Serum AAT level (mg/dl)	96 ± 22^b,c^	<20 ± 0^a,d,e^	<20 ± 0^a,d,e^	113 ± 11^b,c^	109 ± 12^b,c^

Data are mean ± SE. AATD, alpha-1 antitrypsin deficient; AUG, augmentation therapy; PFTnorm, pulmonary function testing normal; AAT, alpha-1 antitrypsin; COPD, chornic obstructive pulmonary disease; DXA, dual-energy x-ray absorptiometry; m, male; f, female; Significance difference between group is denoted as follows, ‘^a^’ different that AATD + AUG, ‘^b^’ different that AATD-AUG, ‘^c^’ different compared to AATD PDTnorm, ‘^d^’ different compared to COPD, ‘^e^’ different compared to Healthy. In all cases, significance is *p* ≤ 0.05.

**Table 2:** Pulmonary function, exercise capacity and smoking history of subject groups

	**AATD + AUG**^**a**^	**AATD-AUG**^**b**^	**AATD PFTnorm**^**c**^	**non-AATD COPD**^**d**^	**Healthy**^**e**^
FVC (liters)	3.85 ± 0.42	2.88 ± 0.16^c^	4.27 ± 0.64^e^	2.80 ± 0.32^c,e^	3.96 ± 0.57^d^
FVC (% pred)	88 ± 6^c^	79 ± 5^c^	109 ± 6^e^	77 ± 8^c,e^	95 ± 6^d^
FEV1 (liters)	1.23 ± 0.25^c,e^	1.35 ± 0.22^c,e^	3.22 ± 0.47^e^	1.49 ± 0.11^c,e^	3.07 ± 0.35^d^
FEV1 (% pred)	35 ± 5^c,e^	47 ± 7^c,e^	107 ± 4^e^	54 ± 7^c,e^	93 ± 4^d^
FEV1/FVC	31 ± 3^c,d,e^	47 ± 7^c,e^	76 ± 7^c,e^	56 ± 4^c,e^	79 ± 2^d^
FEV1/FVC (% pred)	40 ± 4^c,d,e^	61 ± 10^c,e^	97 ± 2^e^	71 ± 6^c,e^	99 ± 3^d^
**Bike (two-legs) exercise**					
Watts	62 ± 6^c,e^	46 ± 11^c,e^	176 ± 47^d,e^	70 ± 11^c,e^	126 ± 17^d^
VO2peak (L/min)	1.02 ± 0.11^c,e^	0.69 ± 0.13^c,e^	2.07 ± 0.51^d^	1.16 ± 0.12^c,e^	1.71 ± 0.25^d^
VO2peak (ml/kg/min)	12.6 ± 0.5^c^	9.5 ± 1.1^c,e^	26.9 ± 5.6^d,e^	14.9 ± 1.7^c^	20.3 ± 3.3
**Knee extensor (single-leg) exercise**					
Watts	25 ± 3^c^	21 ± 7^c^	59 ± 23^d,e^	21 ± 3^c^	26 ± 1
**Smoking history**					
Never	17%	0%	25%	0%	0%
Ex-smoker	83%	100%	75%	43%	100%
Current	0%	0%	0%	57%	0%

Data are mean ± SE. AATD, alpha-1 antitrypsin deficient; AUG, augmentation therapy; PFTnorm, pulmonary function testing normal; AAT, alpha-1 antitrypsin; COPD, chronic obstructive pulmonary disease; FVC, forced vital capacity; FEV1, forced expiratory volume at 1 sec; VO2peak, peak oxygen consumption during exercise. Significance difference between group is denoted as follows, ‘^c^’ different compared to AATD PFTnorm,‘^d^’ different compared to COPD, ‘^e^’ different compared to Healthy. No differences with respect to AATD + AUG ‘^a^’ or AATD-AUG ‘^b^’ were found. In all cases, significance is *p* ≤ 0.05.

All subjects provided verbal and written informed consent prior to participation in the study. The study was approved by the Human Research Protection Program at University of California, San Diego, and was conducted in manner consistent with the Declaration of Helsinki.

### Experimental design and exercise testing

Each individual was studied over a two-day period. On the first day, subjects provided a small blood sample (5 ml via venipuncture) for assessment of hemoglobin concentration, genotyping and measurement of circulating AAT, and baseline concentration of serum for cytokines. Baseline assessment of venous cytokines were obtained on both testing days (Study day 1 and day 2) in order to evaluate what influence, if any, prior exercise testing may have on basal serum cytokines on the following day (i.e. second day of the study). Spirometry was used to assess airway obstruction using a Keystone dry-rolling seal spirometer (S&M Instruments, Louisville, CO, USA). Lean body mass was measured via dual-energy x-ray absorpometry (DXA), and maximal exercise testing was performed using two different exercise modalities, 1) a single-leg knee-extensor exercise (KE), and 2) standard two-legged bicycle exercise. Use of the single-leg KE ergometer has been previously reported by us [37-39] and allows patients with cardiopulmonary disease to perform moderate- to high-intensity muscular work in the presences of limited pulmonary and/or cardiac reserve capacity – allowing assessment of skeletal muscle responses to exercise which otherwise would be severely limited with whole body exercise. Further description of KE exercise and cycle ergometry are provided in Additional file 1.

On Study day 2, subjects had a muscle biopsy and blood sampling performed before and after KE exercise (60 min at 50% of maximal KE determine on day 1). Muscle biopsy from the vastus lateralis muscle (described in Additional file 1) prior to exercise was obtained from the non-exercising leg, while the muscle biopsy post-exercise was obtained 4 hours after the single-leg KE exercise on the exercised leg. Prior to exercise, femoral venous catheter was inserted and secured in the leg to be exercised. Radial arterial catheter was placed in the non-dominant arm. Arterial and venous blood was obtained (in duplicate) at rest, after which samples were obtained at 10 min intervals during the 60 minute KE exercise, followed by blood draws at 30 minute intervals for up to 4-hours post exercise. The 4-hour time point was principally selected based on data demonstrating that peak muscle IL-6 expression occurs between 4–8 hours post exercise [40]. Given that circulating expression of other cytokines (e.g. TNFα and IL-1β) have also been shown to elevated between 1 and 24-hour post exercise [25,40] it was reasoned that examining muscle up to 4 hours post exercise would be an appropriate time to allow muscle levels to be altered.

### Blood sampling and handling

Arterial and venous blood was collected using aseptic technique in serum separator vacutainer tubes (Kendall HealthCare Monoject; Fisher Scientific #22029312) and allowed to clot at room temperature for 20 minutes. Thereafter, blood samples were centrifuged at 3,000 RPM (at 4 C) for 13 minutes. Serum was decanted, aliqouted and stored at −80 C until processed.

### Cytokine assessment

For each cytokine, the venous blood from Study day 2 were compared against pre-exercise venous blood obtained on Study day 1 (screening day) to determine if maximal exercise testing from the previous day significantly altered baseline cytokine concentration on Study day 2. No significant differences in basal expression were observed between venous samples obtained on Study day 1 vs. day 2 (coefficient of variation [CV] for IL-1β = 0.55, IL-6 = 0.50, TNFα = 0.25, CRP = 0.29), nor were differences found among the basal cytokine concentration between duplicate measures of either arterial (average CV for IL-1β = 0.51, IL-6 = 0.35, TNFα = 0.22, CRP = 0.21) or venous blood (average CV for IL-1β = 0.30, IL-6 = 0.41, TNFα = 0.21, CRP = 0.12). Accordingly, the resting arterial and venous values were averaged to yield one resting arterial and venous serum values, respectively, which were used for all further statistical analyses. Additional description of the qualitative assessment of the methodology, variation, and minimal detection levels of the cytokine assays are provided in the Additional file 1: Methods.

### Statistical analyses

Demographic, anthropomorphic, lung function and exercise data were compared across subjects groups using ANOVA with Fisher’s PLSD post hoc testing when significant main effects were observed. Kruskal-Wallis test revealed cytokine data were not normally distributed across the subject groups, therefore cytokine data were square-root transformed and then analyzed using a 2-way mixed effect ANOVA model with repeated measures experimental design using either JMP/Pro V10 (SAS Inst. Inc., Cary, NC) or StatView Analysis (v5.0.01 SASS Inst. Inc., Cary NC). Post-hoc testing was performed when a significant overall *F-*ratio was achieved. Correlations were performed using a Spearman Rank Test. In all cases *p* ≤ 0.05 was used to establish significance. All data shown are mean ± SE, unless otherwise indicated.

## Results

### Subject characteristics

Group data depicting demographic, anthropometric, and serum liver function tests are shown in Table 1. Spirometry data and maximal exercise performance are shown in Table 2. Supplemental tables are also provided to show spirometric comparison of the single MZ subject (Additional file 1: Table S1A) and the SZ subject (Additional file 1: Table S1B) compared to their respective groups. All subjects enrolled completed the study, however one subject (female in the healthy group) was eliminated from the analysis in the study when evidence of undiagnosed pulmonary abnormality was found (i.e. resting arterial PO_2_ = 66 Torr). Healthy subjects were well matched to all patients groups according to age, height and weight (Table 1).

As expected, all patients with COPD had reduced whole body aerobic capacity (i.e. VO2peak) compared to subjects with normal lung function (Table 2). While the healthy subjects had greater aerobic exercise capacity, single-leg knee extensor (KE) exercise capacity was not significantly different compared to COPD patients groups. Total body mass, body mass index (BMI), % body fat and lean body mass were similar between healthy and all the COPD patients, which tends to support the idea that our ‘healthy control group’ subjects were similarly matched based on physical (in)activity.

### Subject group comparisons

**Figure 1:**
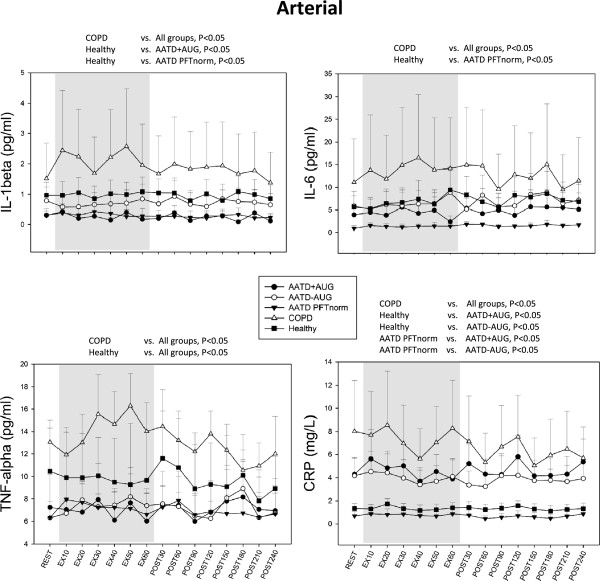
**Arterial serum levels of IL-1β, IL-6, TNFα and CRP obtained under resting conditions (black bar), every 10 minutes during 1-hour single-leg knee extensor exercise at 50% of maximal exercise capacity (gray bars), and every 30 minutes (up to 4 hours) during recovery post exercise (white bars), in COPD individuals with alpha-1 antitrypsin deficiency receiving augmentation therapy (AATD + AUG), AATD individuals not receiving augmentation therapy (AATD-AUG), AATD individuals with normal pulmonary function (AATD PFTnorm), non-AATD COPD patients (COPD), and age-/activity-matched controls with no lung disease (Healthy).** Significant ANOVA main effect for group differences are shown above each graph. In all cases, significance is determined as p < 0.05.

**Figure 2:**
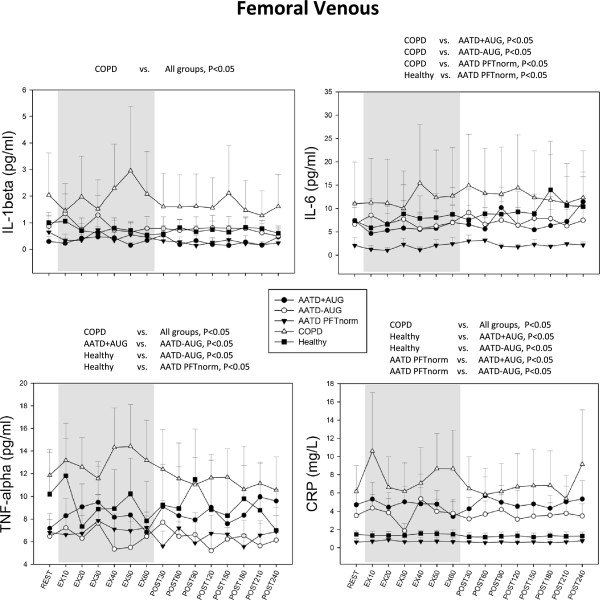
**Femoral venous serum levels of cytokines from same conditions and subject groups in Figure**1**.** Significant ANOVA main effect for group differences are shown above each graph. In all cases, significance is determined as p < 0.05.

Resting serum cytokines were not different for any cytokine in venous blood obtained from Study Day 1 (measured prior to max testing) compared the following Day 2 (resting measured prior to exercise) indicating that previous day exercise activity had no influences on baseline cytokines concentration levels on the following day in our subjects (data not shown).All measured cytokines displayed significant main effects according to subject groups, therefore post hoc testing for group differences were performed for each cytokine. Non-AATD COPD patients exhibited highest circulating concentrations for all cytokines measured (i.e. TNFα, IL-1β, 1 L-6 and CRP) in both arterial and venous blood compared to any subject group (Figures 1 and 2). Expression of all circulating cytokines were significantly greater in non-AATD COPD compared to healthy controls (p < 0.05; Figures 1 and 2), except for venous IL-6 which was not significant (p = 0.099; Figure 2) but displayed a similar trend.In all cases, cytokine concentrations were lower in AATD COPD patients compared to COPD control patients (Figures 1 and 2). Arterial IL-1β and TNFα concentration were also observed to be lower in AATD + AUG patients than in healthy controls (Figure 1). Similarly, both arterial and venous TNFα levels were lower in AATD–AUG compared to healthy controls (Figure 2). In contrast, circulating arterial and venous CRP concentration were elevated in AATD + AUG and AATD–AUG patients compared to healthy controls and AATD PFTnorm, but were significantly lower compared to non-AATD COPD patients (Figures 1 and 2).When comparing AATD COPD individuals ‘on’ versus ‘off’ augmentation therapy (AATD + AUG and AATD–AUG, respectively), we found no differences in the circulating levels of IL-6, IL-1β or CRP (Figures 1 and 2). However, venous TNFα was significantly greater in AATD + AUG compared to AATD–AUG (Figure 2).

### Circulating cytokine response to exercise

**Figure 3:**
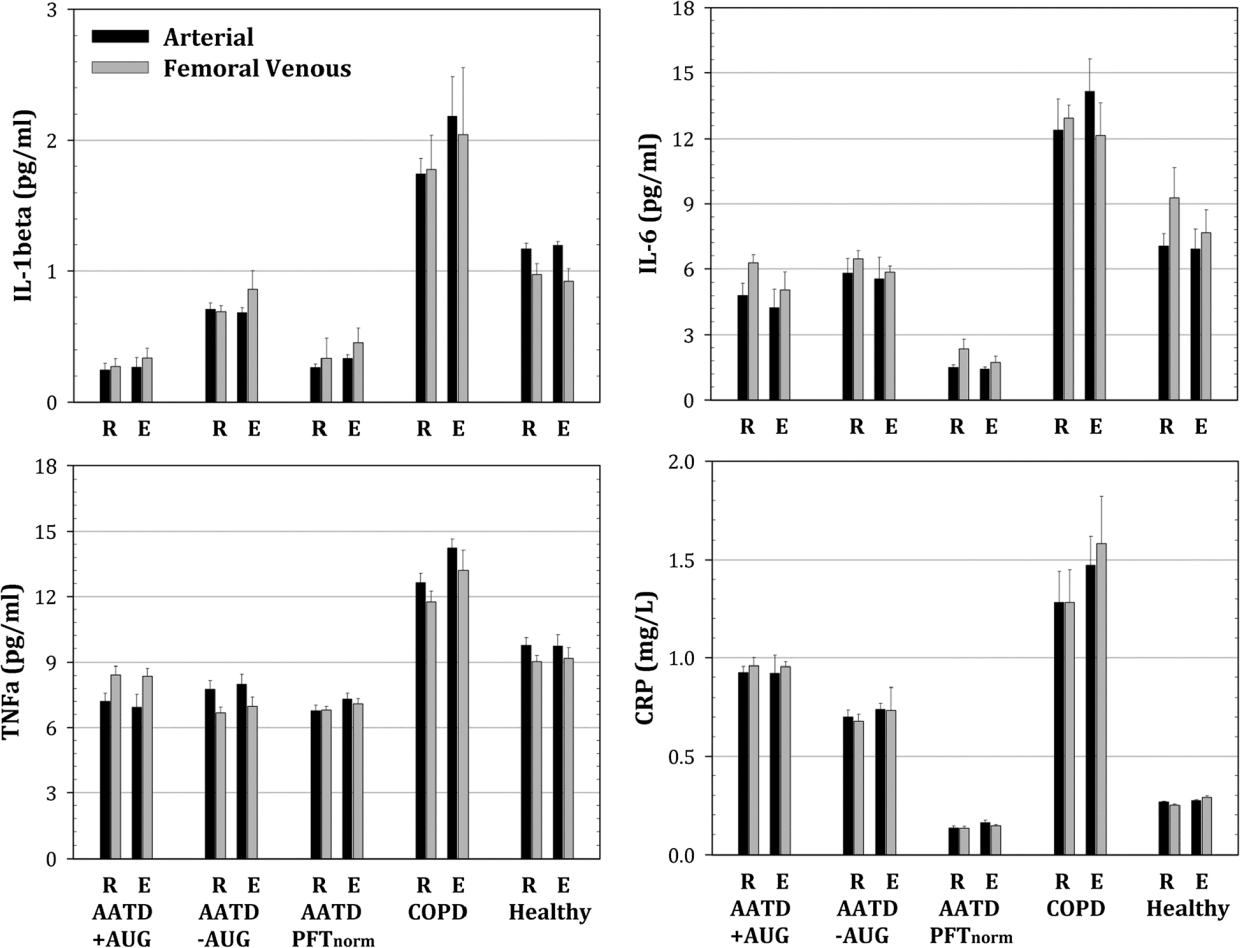
**Summary comparison of arterial (black bars) and femoral venous (gray bars) serum levels for cytokines at rest (R) and during exercise (E) highlighting the observation of, 1) no significant response to exercise and, 2) no significant difference between arterial and femoral venous serum levels among subject groups.** ‘R’ values are averaged data from rest and post-exercise ‘recovery’ time points shown in Figures 1 and 2. ‘E’ values are averaged for all exercise time points shown in Figures 1 and 2. Overall subject group differences are clearly present (as shown in Figures 1 and 2), but statistical notation of these differences are not recapitulate on this summary figure.

Mixed effect ANOVA revealed no significant main effect for exercise (or subject group × exercise interaction) for any of the cytokines measured in arterial blood or venous blood. In addition, a separate mixed effect ANOVA that only included resting and exercise data (i.e. without post-exercise data) revealed no significant main effect for exercise for any of the cytokines measured. A summary depicting the group mean exercise data along with resting cytokine concentrations for both arterial and femoral venous blood is shown in Figure 3.

### Femoral venous-minus-arterial (v-a) difference and skeletal muscle expression

**Figure 4:**
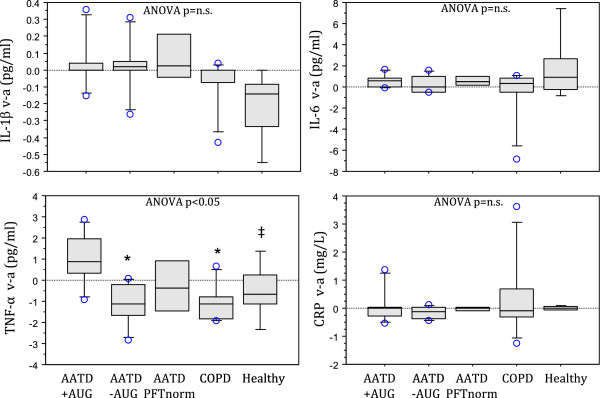
**Box plot showing femoral venous-minus-arterial (v-a) cytokine values obtained from mean venous and arterial data represented for groups in Figures**1**and**2**.** Venous values are obtained from the femoral vein, therefore v-a > 0 imply muscle efflux of cytokines, whereas v-a < 0 imply muscle uptake. Zero v-a values imply no muscle involvement. Box boundaries show 25th and 75th percentile, while whisker bars represent 10th and 90th percentile. All observations >90th percentile are individually represented as open circle (ο) data point. Group median is shown as center line within the box. Post-hoc testing was only performed on TNF-α, where a significant ANOVA main effect (p < 0.05) was observed. * indicates p < 0.05 compared to AATD + AUG, ‡ p = 0.0516 compared to AATD + AUG.

**Figure 5:**
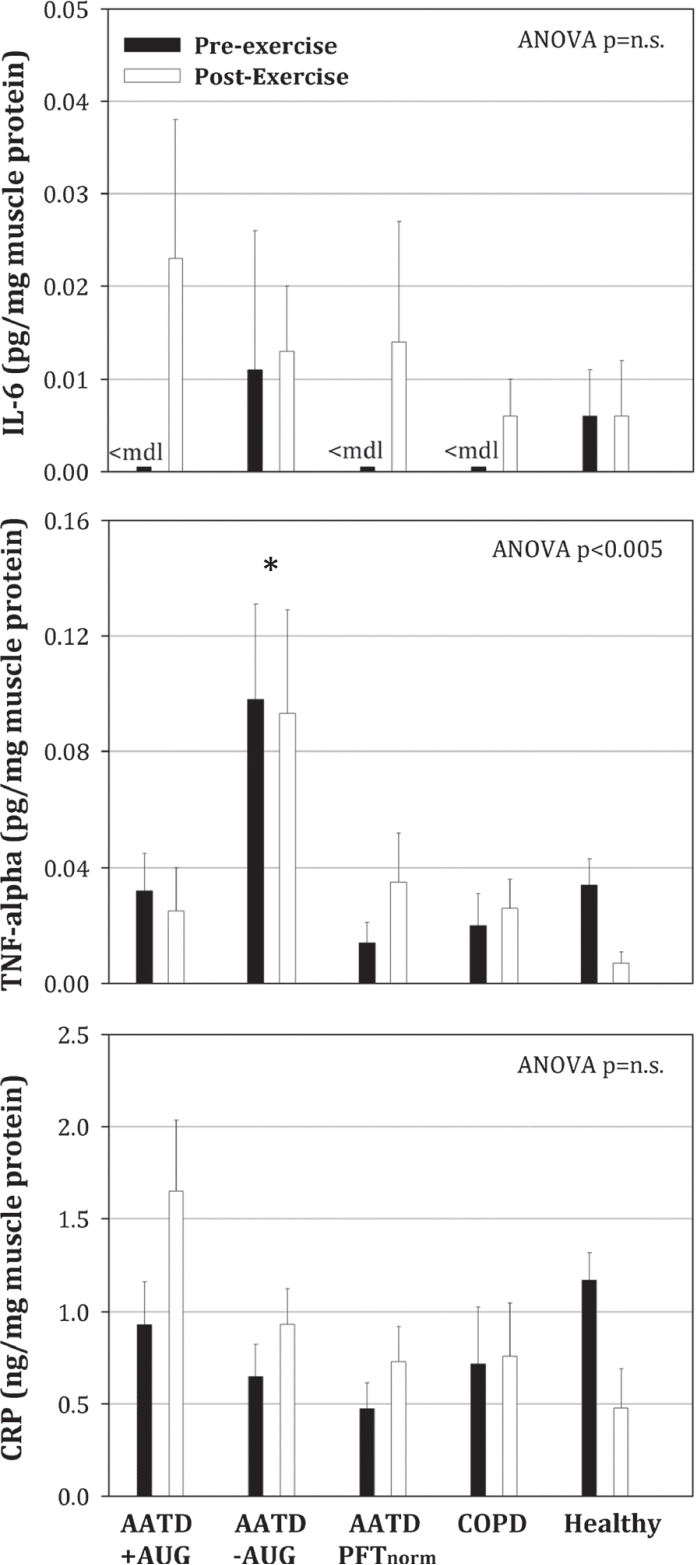
**Skeletal muscle cytokine expression (normalized to total muscle protein) from muscle biopsies of vastus lateralis muscle before and after the 60-minutes of single-leg exercise.** Pre-exercise biopsy was obtained on non-exercising leg prior to exercise, while post-exercise biopsy was obtained 4-hours post-exercise on the exercising leg. We provide indication where detection of cytokines fell below the minimal detectable limit (<mdl) of the assay, i.e. all samples for IL-1b (therefore no data shown) and for some samples in IL-6. Post-hoc testing was only performed on TNFα, where a significant ANOVA main effect (p < 0.005) was observed. No significant main effect was observed for IL-6, therefore post-hoc testing between groups was not performed. * indicates p < 0.05 compared to all other groups.

TNFα was the only cytokine showing significant ANOVA main effect for v-a difference (ANOVA p < 0.05). Post hoc testing revealed TNFα v-a difference was greater in AATD + AUG compared to AATD–AUG and COPD control groups (p < 0.05), along with similar tendency for Healthy subjects (p = 0.0516) (Figure 4).In skeletal muscle, only TNFα was found to exhibit a significant ANOVA main effect (Figure 5). Skeletal muscle TNFα was greater in AATD–AUG subject compared to all other groups (Figure 5). Skeletal muscle expression for IL-1β was below the minimal detectable level of our assay (i.e. 0.08 pg/mL) and therefore unable to be determined.Exercise exerted no significant effect on the skeletal muscle concentration of any cytokines measured (Figure 5).

### Correlation of circulating cytokines to airway obstruction

**Figure 6:**
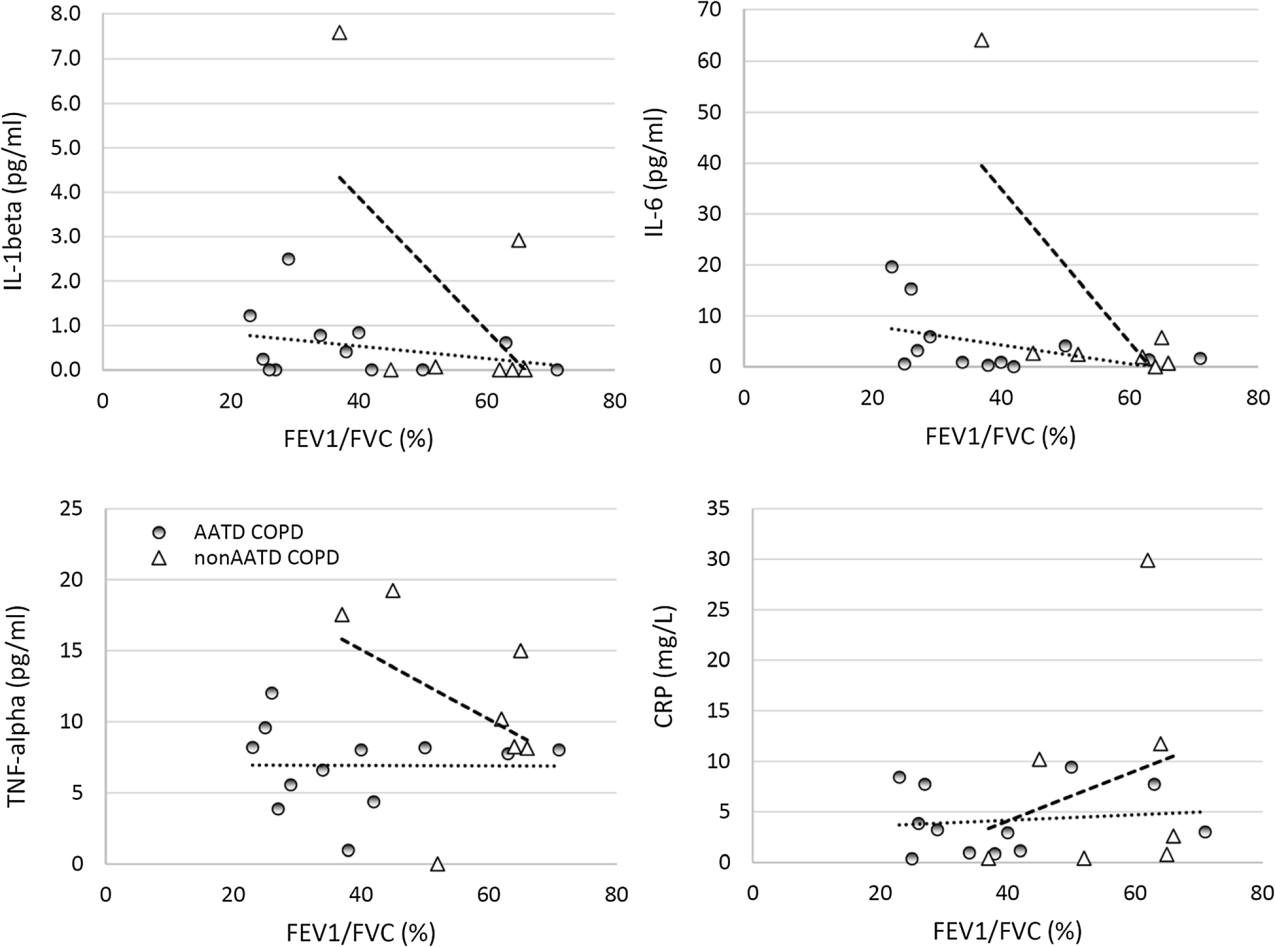
**Correlation between circulating arterial cytokines and airway obstruction for AATD COPD patients (i.e. AATD + AUG and AATD-AUG groups shown as shaded circles) and for non-AATD COPD patients (open triangles).** Dotted regression line reflect data for AATD COPD, whereas dashed regression line reflect data for non-AATD COPD.

In AATD COPD patients (i.e. AATD + AUG and AATD-AUG groups) we find no evidence to support a correlation between circulating arterial cytokines and the degree of airway obstruction (Figure 6). In nonAATD COPD patients, there was a trend for increasing TNFα (p = 0.09), IL-1β (p = 0.57) and IL-6 (p = 0.16) in association with worsening FEV1/FVC, but no significant correlation was observed (Figure 6).

## Discussion

The main findings of this study are, 1) AATD patients with COPD have lower circulating cytokines concentration compared to non-AATD COPD patients, 2) patients with AATD COPD receiving augmentation therapy have lower TNFα expression in skeletal muscle and a greater venous-minus-arterial difference for circulating TNFα (suggesting net efflux of TNFα from the skeletal muscle) compared to AATD COPD patients not receiving augmentation therapy, and 3) 60-minutes of sustained moderate-intensity exercise had no significant effect for any of the measured cytokines. These changes were seen reproducibly across sequential arterial and venous blood samples within individual subjects and across patient subject groups.

### Systemic inflammation in AATD and non-AATD COPD

Large scale cross-sectional studies have documented the association between systemic inflammation and reduced lung function in patients with COPD [41] as well as in healthy subjects [42]. Our data, showing elevated circulating concentrations of IL-1β, IL-6, TNFα and CRP are consistent with both clinical and laboratory evidence showing increased chronic systemic inflammation in COPD. More importantly, our data show that this finding cannot be generalized to patients with COPD resulting from AATD. In all cases, cytokine concentration was lower in AATD individuals (with or without lung disease) compared to the non-AATD COPD patients (Figures 1 and 2). When compared to healthy control subjects, only CRP was elevated in AATD COPD patients, but this increase was still lower compared to CRP in non-AATD COPD patients (Figures 1, 2 and 3).

The elevated CRP concentration seen in our AATD subjects with COPD may lend support to the idea that polymers from abnormal variants of α_1_-antitrypsin alleles are pro-inflammatory [43-45]. However, since AATD individuals with normal spirometry (AATD_PFTnorm_) did not have elevated CRP, and yet exhibit a similar low circulating AAT as AATD-AUG with COPD (Table 1), it seems more likely the elevated CRP is associated with the pathophysiology of the COPD sequelae rather than a response to polymers associated with α_1_-antitrypsin allele variants. It is surprising, however, that AATD COPD patients had lower blood levels of CRP (compared to non-AATD COPD patients, Figures 1, 2 and 3) but yet exhibited similar, if not greater, airway obstruction (Table 1). Based on the existing literature [46,47], it might have been expected that individuals with the worst lung function would have the greatest increases in circulating CRP. One explanation may be that plasma CRP is produced principally in hepatocytes and individuals with AATD (in addition to developing emphysema) can also develop cirrhosis of the liver, potentially leading to liver failure as a result of excess deposition of abnormal α_1_-antitrypsin protein in the liver. But we found no evidence of liver dysfunction in any of AATD subjects (Table 1). There are only few small studies that have evaluated the risk of cardiovascular events in AATD individuals [48-50]. Despite conflicting results [51], there is evidence of altered vessel elasticity (e.g. increased aortic wall stiffness) that supports elevated risk of cardiovascular disease [49,50], as well as occult musculoskeletal changes [48], both of which could contribute to the morbidity and mortality in AATD individuals. Although the severity of pulmonary dysfunction appears worse in AATD patients with COPD (compared to non-AATD COPD patients), circulating biomarkers for inflammatory cytokines (other than CRP) in AATD COPD patients were similar to healthy subjects. These data highlight the fact that it cannot be assumed that the inflammatory sequelae for COPD are similar between AATD and non-AATD COPD patients. Indeed, while non-AATD COPD patients are often found to have elevated circulating TNFα concentration [see meta-analysis review, Ref [41]], as seen in our data (Figures 1 and 2), we find no significant evidence of elevated circulating TNFα in AATD COPD individuals - even with greater degree of airway obstruction (Figure 6). Previous studies examining inflammatory biomarkers in PiZZ COPD patients report similar observations [9,52]. Collectively, these data oppose the idea that systemic inflammation contributes to the underlying lung pathology in patients with AATD COPD, as AATD COPD individuals with the greatest deficits in lung function exhibited minimal evidence of systemic inflammation. These data also add to the recognition that while the clinical manifestations of COPD are often very similar, the underlying mechanism and pathology are much less easily defined or even homogeneous in nature. It is important for future COPD research to evaluate the origins of the heterogeneity observed in patients with COPD before we can better understand how best to treat and individually manage patients suffering from COPD.

### Effect of augmentation therapy on inflammatory cytokines

In general we found no significant differences between AATD patients with COPD receiving augmentation therapy (AATD + AUG) compared to those not receiving augmentation therapy (AATD–AUG). However, it was observed that AATD + AUG patients had slightly greater circulating TNFα in venous blood compared to AATD–AUG patients (Figure 2). A significant difference in circulating TNFα between these groups was also seen when calculating the venous-minus-arterial difference in TNFα (Figure 4). The latter finding (i.e. positive TNFα v-a difference due to greater femoral venous versus arterial concentration) suggests that there is net outflow or ‘spillover’ of TNFα into the circulation from skeletal muscle in AATD + AUG patients. In contrast, the negative TNFα v-a values (i.e. higher arterial versus femoral venous concentrations) seen in AATD–AUG subjects (Figure 4), suggests a net muscle uptake of TNFα in these subjects. This finding is supported by elevated TNFα expression in skeletal muscle of AATD–AUG subjects that is not seen in AATD + AUG subjects (Figure 5). However, the interpretation of this finding still remains somewhat ambiguous, given that similar negative TNFα (v-a) values were seen in the control groups (i.e. AATD_PFTnorm_, non-AATD COPD, and Healthy), yet only AATD–AUG subjects exhibited elevated skeletal muscle TNFα.

CRP was the only other cytokine found to be greater in AATD patients with COPD compared to controls (as previously discussed). But for CRP there was no difference between AATD individuals ‘on’ or ‘off’ augmentation therapy, suggesting that AAT replacement therapy had no effect on circulating CRP concentration. Taken together, it remains uncertain what clinical effect, if any, that differences in circulating TNFα and CRP may actually exert between AATD patients ‘on’ versus ‘off’ augmentation therapy.

### Inflammatory response in skeletal muscle

Several studies have reported on the cytokine responses in skeletal muscle of COPD patients. [53-55], but to our knowledge no studies have examined skeletal muscle in AATD individuals or what effect augmentation therapy may have. Our data suggest that there is elevated TNFα in the skeletal muscle of AATD–AUG subjects (Figure 5), which, to some extent, supports the implication of an active musculoskeletal inflammatory process. But, as previously mentioned for circulating cytokines, the importance of this finding remains somewhat unclear in muscle, since 1) increases in other proinflammatory cytokines were not observed in the skeletal muscle of this patient group, and 2) a similar increase in muscle TNFα was not also observed in the non-AATD COPD patient group who also had evidence of elevated systemic inflammation. But, whether or not TNFα is typically elevated in skeletal muscle of patients with COPD remains controversial. While some studies report elevated muscle TNFα expression [28,54], others show no change [56,57] or decreased TNFα [53]. These discrepancies might be explained by heterogeneity in patient population (as we show), by differences in study design, and/or in the sensitivity of the assay(s) that are used to measure TNFα.

### Exercise and inflammation

In healthy individuals, strenuous exercise elicits an acute phase inflammatory response that is rapidly counter-balanced by anti-inflammatory cytokines [25]. This acute phasic response is actually beneficial and helps to maintain a strong and healthy immune system. But there has been concern that habitual exercise in COPD patients may superimpose an added inflammatory burden that may prove more harmful than good [58]. Our data, using moderate-intensity exercise (for 1-hour) at approximately 50% of individual’s maximal aerobic power, finds no evidence of exercise-induced inflammatory stress in either AATD patients (with no underlying systemic inflammation) or non-AATD COPD patients (which had evidence of chronic systemic inflammation). This is consistent with other data reporting acute inflammatory responses to exercise in patients with COPD [59], and more generally with large-scale epidemiologic studies that have repeatedly established the benefits of exercise in maintaining health and longevity. Indeed, exercise capacity is touted to be the best predictor of an AATD individual’s health status (better than high-resolution computed tomography or lung function assessments) [60], and our data finds no evidence to suggest that exercise activates or exacerbates either acute or chronic inflammatory pathways in either AATD or non-AATD COPD patients. To the contrary, AATD individuals with normal spirometry had far greater functional exercise capacity (seen with both cycle ergometry and KE exercise, Table 1), which could suggest that maintaining one’s aerobic capacity may be protective for COPD progression. However, based on our study design, we cannot know whether aerobic exercise is truly protective for disease progression, or if this observation is simply a coincidence based on the small subset of the individuals in our study.

### Study limitations

When making cross-sectional group comparisons, it must be recognized that our relatively small subject population (n = 4–7 subjects per group) combined with multiple comparisons could increase the potential of identifying spurious relationships. But, our study also included a repeated measure study design, in which each subject served at their own control. Indeed, from each subject we obtained 34 temporally independent blood measurements (17 arterial, 17 femoral venous) and 2 muscle biopsy measurements (one pre-exercise and one post-exercise). Given the number of individual measurements made in each subject, the statistical power for the repeated measures relating to exercise response is high (i.e. β >0.95). Based on the large number of individual blood measurements made, we also have a high degree of confidence in the individual mean values obtained for each subject. While this does not eliminate the concern for variability among small total number of subjects within each group (i.e. the cross-group comparisons), there is greater confidence in the accuracy of the absolute mean values for each subject that helps to increase confidence in the interpretation of the cross-sectional data. For example, comparing variability in Figures 1 and 2 (which report individual rest and exercise data points) to Figure 3 data (which reports a summary of mean values for rest and exercise data, respectively) it is clear the lower variability evident in Figure 3 provides greater confidence in the evaluating across patient groups differences that are mirrored in Figures 1 and 2. Nonetheless, it must be stated, given the small number of patients in each group, any cross-sectional interpretation must be view with caution.

## Conclusion

These data establish that AATD patients with COPD do not experience the same inflammatory cytokine response as that typically seen in non-AATD COPD patients. However, it is evident the circulating the CRP (albeit lower compared to that seen in non-AATD COPD patients) is elevated in AATD COPD patients compared to controls subjects with normal lung function. In general, augmentation therapy did not appear to alter circulating cytokine concentrations, except for TNFα. AATD COPD patients on augmentation therapy exhibited a positive venous-minus-arterial TNFα serum difference, whereas those off augmentation therapy exhibited a negative venous-minus-arterial TNFα difference. This negative v-a difference (suggesting muscle sequestration of TNFα) in AATD COPD subjects not receiving augmentation therapy correlated with significantly higher muscle concentration of TNFα compared to patients on augmentation therapy. While these data cannot establish a cause-and-effect relationship, they suggest that patients receiving augmentation therapy may have net efflux of TNFα from the skeletal muscle. Lastly, our data indicate that exercising for 60 minutes at 50% of maximal work capacity does not significantly increase expression of circulating inflammatory cytokines. Collectively, these data suggest that systemic inflammation is not simply a “spill over” function of pathophysiology associated with destruction of lung tissue *per se*, but rather suggests that compounds or mechanism(s) associated with environmental risk factors (such as tobacco smoke) likely play a principle role in eliciting the inflammation reaction.

## Competing interests

Funding for this project was supported by an unrestricted grant from CSL Behring. Support was also provided by the UCSD General Clinical Research Center in conducting DXA scanning. Dr. Olfert received funding support from American Heart Association (10BGIA3630002), UCSD National Skeletal Muscle Research Group (NIH R24HD050837), West Virginia University School of Medicine and West Virginia Clinical and Translational Science Institute (NIH/NIGMS U54GM104942). Support for Drs. Wagner and Malek were provided NIH grant P01 HL17731-28. Funding for WVU Core Facilities used for cytokine analyses was provided by NIH grants P30 GM103488 and P30 RR032138.

None of the authors have any personal financial support or involvement with CSL Behring. Study support from CSL Behring did not influence study design, data collection, analysis or interpretation, writing of the manuscript or decision to submit for publication.

## Authors’ contributions

IMO and PDW conceived of the study design and were involved in all aspects for the study (i.e. patient recruitment, data collection, analysis, and manuscript preparation). MHM, TMLE and HW were involved in providing intellectual content, data collection, data analysis, and drafting/editing the manuscript. All authors read and approved the final manuscript.

## Pre-publication history

The pre-publication history for this paper can be accessed here:

http://www.biomedcentral.com/1471-2466/14/106/prepub

## Supplementary Material

Additional file 1 Supplemental Material. Click here for file
