# Supplementary material for: Inflammatory cytokine response to exercise in alpha-1-antitrypsin deficient COPD patients ‘on’ or ‘off’ augmentation therapy
Source: BMC Pulm Med. 2014 Jun 30;14:106. doi: 10.1186/1471-2466-14-106 (PMC4091645; doi:10.1186/1471-2466-14-106)
Supplement: Additional file 1 — Supplemental Material. [file 1471-2466-14-106-S1.docx]

**Additional file 1: SUPPLEMENTAL MATERIAL**

**METHODS**

**Maximal Exercise Testing**

Two-legged cycle ergometry was performed in the standard manner using an electronically braked ergometer (Lode Excaliber Sport, Quinton Instruments Inc., Groningon). Knee extensor (KE) exercise was performed with the subjects seated in an adjustable car seat that is attached to a specially adapted Monark cycle ergometer behind the seat. The subject’s ankle from one leg is placed in a rigid boot attached to an aluminum bar connected to the crank arm of the ergometer, resulting in isolated exercise to the quadriceps muscle. During the KE exercise, subjects were asked to maintain 60 RPM using visual feedback provided by an electronic RPM meter [1-3].

For each maximal test, subjects were allowed to warm up (unloaded kicking/pedaling) and then performed an incremental exercise test to the limit of tolerance, in which work intensity was increased every 1 min (3-5 watts for KE, 5-25 watts for two-leg cycling) such that exhaustion would occur between 10-15 minutes. The test order was KE exercise followed bipedal cycling with a minimum of 1-hour between the two maximal exercise tests for the subjects to rest.

**Muscle Biopsy**

Percutaneous muscle biopsies (i.e. one at rest in the non-exercising leg, and the second 4-hours after completion in KE exercised in the exercising leg) were obtained from the vastus lateralis muscle from ventral midline between the hip and knee using a Bergstrom needle at an approximate depth of 3-5 cm. Upon obtaining biopsies, muscle samples were immediately weighed and flash frozen in liquid N_2_. All samples were stored at -80 C until process for cytokine analyses.

For analysis, ~100 mg of muscle from each subject was separately homogenized in a lysis buffer containing 50 mM Tris/Hcl (pH 7.4), 150 mM NaCl, 0.5% Triton X-100, and protease inhibitors (Complete™ Tablet, Roche Company, Mannheim, Germany). Homogenates were centrifuged at 4°C, 10,000 rpm, for 10 minutes and supernatants assayed for total protein concentration using the Bradford Assay (Coomassie Plus #23236 Assay Pierce Biotechnology, Rockford, IL). Muscle supernatant was run undiluted on Luminex analysis platform and diluted 1:200 with assay dilute for C-Reactive Protein (CRP) analysis as per manufacture recommendations (*instrument details provided below*).

**Qualitative determination of cytokine assays.**

Cytokine analyses of serum and skeletal muscle homogenate for TNFα, IL-6 and IL-1β was performed using a multiplex Human Cytokine Magnetic-bead based assay (HSCYTMAG-60SK-03, EMD Millipore, Saint Charles, MO) on a Luminex200 system (Luminex Corporation, Austin, TX). C-reactive protein (CRP) concentration was measured using a human high-sensitivity assay kit according to manufacturer protocol (K151EPC, MesoScale Diagnostics, LLC, Gaithersburg, MD) on a SECTOR Imager2400 Instrument (MesoScale Diagnostics, LLC, Gaithersburg, MD). Each subject samples were run in duplicate on a single plate and then averaged for final analysis.

The average coefficient of variation of the standard curves generated compared to the expected standard values was found to be 1.24±0.54% (mean±SE) for IL-1β, 0.88±0.10% for IL-6, 1.09±0.18% for TNFα and 3.9±0.3% for CRP. Mimimal detection levels for cytokines we as follows: TNFα = 0.7 pg/ml , IL-6 = 0.9 pg/ml, IL-1β = 0.8 pg/ml, and hsCRP = 0.008 ng/ml.

**SUPPLEMENTAL RESULTS
SUPPLEMENTAL TABLE 1A**. Comparison of healthy MZ subject to the remainder of the Healthy subjects shown in Table 1.

|  |  | **MZ subject, n=1** | **MM subjects in Healthy Group, n=4** | | |
| --- | --- | --- | --- | --- | --- |
|  |  |  | **Ave** | **SD** | **SE** |
| **Sex** |  | Male | 2 Male, 2 Female | |  |
| **Age** | *yr* | 58 | 56 | 1 | 1 |
| **Height** | *cm* | 173 | 172 | 12 | 6 |
| **Body Mass** | *kg* | 84 | 86 | 26 | 13 |
| **DEXA Body Fat** | *%* | 27% | 34% | 11% | 6% |
| **Lean Body Mass** | *kg* | 61.6 | 56.5 | 16.9 | 8.4 |
| **Body Mass Index** | *kg/m2* | 28.2 | 28.7 | 5.4 | 2.7 |
| **Fat-Free Mass Index** | *kg/m2* | 20.6 | 18.8 | 3.1 | 1.5 |
| **Lean Body Mass (DEXA)** | *kg* | 57.5 | 53.2 | 14.1 | 7.1 |
| **Thigh Mass (DEXA)** | *kg* | 17.2 | 15.6 | 3.9 | 2.0 |
| **FVC** | *L* | 4.04 | 3.94 | 1.47 | 0.73 |
| **FVC % pred** | *%* | 90 | 96 | 16 | 8 |
| **FEV1** | *L* | 3.34 | 3.01 | 0.90 | 0.45 |
| **FEV1 % pred** | *%* | 94 | 93 | 10 | 5 |
| **FEV1/ FVC** | *%* | 82.0 | 77.8 | 5.7 | 2.9 |
| **FEV1/ FVC % pred** | *%* | 104 | 98 | 7 | 3 |
| **Bike VO2max** | *L/min* | 2.28 | 1.56 | 0.54 | 0.27 |
|  | *ml/kg/min* | 27 | 19 | 7 | 4 |
|  | *watts* | 130 | 125 | 45 | 23 |

**SUPPLEMENTAL TABLE 1B**. Comparison of AATD-AUG SZ subject to the remainder PiZZ subjects in the AATD-AUG group shown in Table 1.

|  |  | **SZ subject, n=1** | **ZZ subjects AATD-AUG group, n=5** | | |
| --- | --- | --- | --- | --- | --- |
|  |  |  | **Ave** | **SD** | **SE** |
| **Sex** |  | Male | 1 Male, 4 Female | |  |
| **Age** | *yr* | 61 | 64 | 12 | 6 |
| **Height** | *cm* | 188 | 167 | 5 | 2 |
| **Body Mass** | *kg* | 146 | 71 | 14 | 6 |
| **DEXA Body Fat** | *%* | 38% | 37% | 9% | 4% |
| **Lean Body Mass** | *kg* | 91.0 | 44.1 | 6.7 | 4.8 |
| **Body Mass Index** | *kg/m2* | 41.3 | 25.5 | 4.8 | 2.2 |
| **Fat-Free Mass Index** | *kg/m2* | 25.7 | 15.8 | 2.1 | 0.9 |
| **Lean Body Mass (DEXA)** | *kg* | 83.3 | 44.7 | 7.3 | 3.3 |
| **Thigh Mass (DEXA)** | *kg* | 24.1 | 11.3 | 1.6 | 0.7 |
| **FVC** | *L* | 3.40 | 2.78 | 0.34 | 0.15 |
| **FVC % pred** | *%* | 64 | 82 | 9 | 4 |
| **FEV1** | *L* | 2.13 | 1.19 | 0.41 | 0.18 |
| **FEV1 % pred** | *%* | 52 | 46 | 19 | 8 |
| **FEV1/ FVC** | *%* | 63.0 | 43.8 | 17.5 | 7.8 |
| **FEV1/ FVC % pred** | *%* | 81 | 57 | 24 | 11 |
| **KE Leg VO2max** | *L/min* | 1.55 | 0.57 | 0.21 | 0.09 |
|  | *ml/kg/min* | 11 | 7.82 | 2 | 1 |
|  | *watts* | 55 | 14 | 5 | 2 |

SUPPLEMENTAL REFERENCES

1. Richardson R, Leek B, Gavin T, Haseler L, Mudaliar S, Henry R, Mathieu-Costello O, Wagner P: **Reduced mechanical efficiency in chronic obstructive pulmonary disease but normal peak VO2 with small muscle mass exercise**. *Am J Respir Crit Care Med* 2004, **169**:89-96.

2. Esposito F, Mathieu-Costello O, Shabetai R, Wagner PD, Richardson RS: **Limited maximal exercise capacity in patients with chronic heart failure: partitioning the contributors**. *J Am Coll Cardiol* 2010, **55**(18):1945-1954.

3. Esposito F, Reese V, Shabetai R, Wagner PD, Richardson RS: **Isolated quadriceps training increases maximal exercise capacity in chronic heart failure: the role of skeletal muscle convective and diffusive oxygen transport**. *J Am Coll Cardiol* 2011, **58**(13):1353-1362.
